# Supplementary material for: Plant species diversity assessment and monitoring in catchment areas of River Chenab, Punjab, Pakistan
Source: PLoS One. 2022 Aug 12;17(8):e0272654. doi: 10.1371/journal.pone.0272654 (PMC9374230; doi:10.1371/journal.pone.0272654)
Supplement: S1 Table — (DOCX) [file pone.0272654.s001.docx]

**S1 Table Checklist of Floral Diversity in order to Botanical Names, Families, Habits and Groups from the Study Area**

| S. No. | Family | Botanical name | Voucher No. | Habit | Group |
| --- | --- | --- | --- | --- | --- |
| 1 | Acanthaceae | *Adhatoda vasica* L. | UOG-000323 | Shrub | Dicot |
| 2 | Amaranthaceae | *Alternanthera pungens* Kunth. | UOG-000320 | Herb | Dicot |
|  |  | *Achyranthes aspera* L. | UOG-000317 | Herb | Dicot |
|  |  | *Digera muricata* L. | UOG-000479 | Herb | Dicot |
|  |  | *Digera arvensis* Forsk. | UOG-000319 | Herb | Dicot |
| 3 | Apiaceae | *Anethum graveolens* L. | UOG-000460 | Herb | Dicot |
|  |  | *Scandix stellata* Banks & Soland. | UOG-000324 | Herb | Dicot |
| 4 | Apocynaceae | *Calotropis procera* Ait. | UOG-000322 | Shrub | Dicot |
| 5 | Araceae | *Pistia stratiotes* L. | UOG-000405 | Herb | Dicot |
|  |  | *Spirodela polyrhiza*L. | UOG-000404 | Herb | Dicot |
| 6 | Asphodelaceae | *Asphodelus fistulosus* L*.* | UOG-000456 | Herb | Dicot |
| 7 | Asteraceae | *Lactuca serriola* L. | UOG-000487 | Herb | Dicot |
|  |  | *Artemisia absinthium* L. | UOG-000467 | Herb | Dicot |
|  |  | *Silybum marianum* L. | UOG-000312 | Herb | Dicot |
|  |  | *Artemisia capilaris* Thunb. | UOG-000457 | Herb | Dicot |
|  |  | *Cichorium intybus* L. | UOG-000469 | Herb | Dicot |
|  |  | *Cyothocline purpera* L. | UOG-000472 | Herb | Dicot |
|  |  | *Conyza Canadensis* L. | UOG-000314 | Herb | Dicot |
|  |  | *Cirsium arvense* L. | UOG-000420 | Herb | Dicot |
|  |  | *Inula hirta* L. | UOG-000484 | Herb | Dicot |
|  |  | *Xanthium strumarium* L. | UOG-000306 | Shrub | Dicot |
|  |  | *Parthenium hysterophorus* L. | UOG-000311 | Shrub | Dicot |
| 8 | Azioceae | *Trianthema portulacastrum* L. | UOG-000301 | Herb | Dicot |
| 9 | Bonaginaceae | *Arnebia hispidissima* (Sieber ex Lehm.) DC. | UOG-000459 | Herb | Dicot |
|  |  | *Cordia domestica* Roth. | UOG-000468 | Tree | Dicot |
|  |  | *Lithospermum arvense* L. | UOG-000486 | Herb | Dicot |
| 10 | Brassicaceae | *Capsella bursa-pastoris* L. | UOG-000425 | Herb | Dicot |
|  |  | *Nasturtium officinale* Ait. | UOG-000497 | Herb | Dicot |
|  |  | *Sisymbrium irio* L. | UOG-000507 | Shrub | Dicot |
| 11 | Canabaceae | *Cannabis sativa* L. | UOG-000336 | Shrub | Dicot |
| 12 | Chenopodiacae | *Chenopodium murale* L. | UOG-000330 | Herb | Dicot |
|  |  | *Chenopodium album* L. | UOG-000329 | Herb | Dicot |
| 13 | Commelinaceae | *Commelina diffusa* Burman f. | UOG-000477 | Herb | Dicot |
| 14 | Companulaceae | *Triodanis perfoliata* L. | UOG-000515 | Shrub | Dicot |
| 15 | Convolvulaceae | *Convolvulus arvensis*L. | UOG-000334 | Herb | Dicot |
| 16 | Cryophyllaceae | *Silene conica* L. | UOG-000509 | Herb | Dicot |
|  |  | *Stellaria media* L. | UOG-000427 | Herb | Dicot |
|  |  | *Setaria pumila* Roem. & Schult*.* | UOG-000510 | Herb | Dicot |
| 17 | Cyperaceae | *Carex praegracilis* W. Boott. | UOG-000471 | Herb | Monocot |
|  |  | *Cyperus diffuses* Vahl. | UOG-000473 | Herb | Monocot |
|  |  | *Cyperus rotundus* L. | UOG-000337 | Herb | Monocot |
|  |  | *Cyperus esculentus* L. | UOG-000474 | Herb | Monocot |
|  |  | *Cyperus elegans* L. | UOG-000475 | Herb | Monocot |
| 18 | Drypoteridaceae | *Polystichum munitum* C. Presl. | UOG-000503 | Herb | Pteridophytes |
| 19 | Equiteceae | *Equisetum arvensis* L. | UOG-000400 | Herb | Pteridophytes |
| 20 | Euphorbiaceae | *Chrozophora tinctoria* L. | UOG-000470 | Herb | Dicot |
|  |  | *Croton bonplandianus* L. | UOG-000476 | Herb | Dicot |
|  |  | *Euphorbia prostrata* Ait. | UOG-000342 | Herb | Dicot |
|  |  | *Euphorbia hirta* L. | UOG-000341 | Herb | Dicot |
|  |  | *Ricinus communis* L. | UOG-000345 | Shrub | Dicot |
| 21 | Fabaceae | *Acacia arabica* Lam. | UOG-000463 | Tree | Dicot |
|  |  | *Acacia nilotica* L. | UOG-000464 | Tree | Dicot |
|  |  | *Alhagi maurorum* Medik. | UOG-000465 | Shrub | Dicot |
|  |  | *Albizia lebbeck* L. | UOG-000466 | Tree | Dicot |
|  |  | *Cassia occidentalis* L. | UOG-000478 | Shrub | Dicot |
|  |  | *Dalbergia sissoo Roxb.* | UOG-000448 | Tree | Dicot |
|  |  | *Ipomoea carnea* Jacq. | UOG-000335 | Shrub | Dicot |
|  |  | *Leucaena leucocephala* Lam. | UOG-000485 | Herb | Dicot |
|  |  | *Lathyrus aphaca* L. | UOG-000348 | Herb | Dicot |
|  |  | *Medicago sativa* L. | UOG-000491 | Herb | Dicot |
|  |  | *Medicago polymorpha* L. | UOG-000492 | Herb | Dicot |
|  |  | *Melilotus albus* Medik. | UOG-000493 | Herb | Dicot |
|  |  | *Trifoliumindicum*L. | UOG-000513 | Herb | Dicot |
|  |  | *Trifolium resupinatum* L. | UOG-000514 | Herb | Dicot |
|  |  | *Vicia sativa* L. | UOG-000517 | Herb | Dicot |
| 22 | Geraniaceae | *Geranium rotundifolium* L. | UOG-000483 | Herb | Dicot |
| 23 | Lamiaceae | *Lamium amplexicaule* L. | UOG-000488 | Herb | Dicot |
|  |  | *Leucas aspera* Willd. | UOG-000354 | Herb | Dicot |
|  |  | *Leucas zeylanica* L. | UOG-000489 | Herb | Dicot |
|  |  | *Teucrium lamiifolium* d'Urv. | UOG-000512 | Herb | Dicot |
| 24 | Malvaceae | *Alyogyne hakeifolia* R. Br. | UOG-000462 | Herb | Dicot |
|  |  | *Malvastrum coromandelianum* L. | UOG-000490 | Herb | Dicot |
| 25 | Marcantiaceae | *Marchantia polymorpha* L. | UOG-000415 | Herb | Bryophytes |
| 26 | Marcilaaceae | *Marsilea quadrifolia* L. | UOG-000403 | Herb | Pteridophytes |
| 27 | Mazaceae | *Mazus reptans* N.E.Br. | UOG-000495 | Herb | Dicot |
| 28 | Moraceae | *Broussonetia papyrifera* L. | UOG-000362 | Tree | Dicot |
|  |  | *Ficus palmate* Forsk. | UOG-000482 | Tree | Dicot |
|  |  | *Ficus religiosa* L. | UOG-000364 | Tree | Dicot |
|  |  | *Morus nigra* L. | UOG-000367 | Tree | Dicot |
|  |  | *Morus alba* L. | UOG-000366 | Tree | Dicot |
|  |  | *Ficus tinctoria* G.Forst. | UOG-000494 | Tree | Dicot |
| 29 | Myrtaceae | *Eucalyptus globulus* Labill. | UOG-000368 | Tree | Dicot |
|  |  | *Eucalyptus camaldulensis* Dehnh. | UOG-000369 | Tree | Dicot |
| 30 | Nelumboaceae | *Nelumbo nucifera* Gaertn. | UOG-000496 | Herb | Dicot |
| 31 | Onagnaceae | *Oenothera rosea* Ait. | UOG-000498 | Herb | Dicot |
| 32 | Osmundaceae | *Osmunda regalis* L*.* | UOG-000401 | Herb | Pteridophytes |
| 33 | Oxalidaceae | *Oxalis corniculata* L. | UOG-000372 | Herb | Dicot |
| 34 | Papavaraceae | *Argemone Mexicana* L. | UOG-000458 | Herb | Dicot |
| 35 | Phyllanthaceae | *Phyllanthus niruri* L. | UOG-000504 | Herb | Dicot |
| 36 | Poaceae | *Agrostis nebulosa* Boiss. & Reut. | UOG-000461 | Herb | Monocot |
|  |  | *Cynodon dactylon* L. | UOG-000382 | Herb | Monocot |
|  |  | *Dichanthium annulatum* Forsk. | UOG-000440 | Herb | Monocot |
|  |  | *Eragrostis minor Host.* | UOG-000480 | Herb | Monocot |
|  |  | *Urena lobata*L. | UOG-000481 | Herb | Monocot |
|  |  | *Eleusine indica* L. | UOG-000380 | Herb | Monocot |
|  |  | *Physalis minor* L. | UOG-000443 | Herb | Monocot |
|  |  | *Poa annua*L. | UOG-000379 | Herb | Monocot |
|  |  | *Phragmites karka* Adans. | UOG-000506 | Herb | Monocot |
|  |  | *Saccharum spontaneum* L. | UOG-000375 | Herb | Monocot |
| 37 | Polygonaceae | *Polygonum plebeium* R.Br. | UOG-000499 | Herb | Dicot |
|  |  | *Polygonum aviculare* L. | UOG-000500 | Herb | Dicot |
| 38 | Pontedariaceae | *Pontedaria crassipes* Mart. | UOG-000410 | Herb | Dicot |
| 39 | Pteridaceae | *Adiantum capillus-veneris* L. | UOG-000402 | Herb | Pteridophytes |
| 40 | Ranunculaceae | *Ranunculus muricatus* L. | UOG-000391 | Herb | Dicot |
| 41 | Rhamnaceae | *Ziziphus jujube* Mill. | UOG-000390 | Tree | Dicot |
| 42 | Rosaceae | *Potentilla argentea* L. | UOG-000501 | Herb | Dicot |
| 43 | Salicaceae | *Populus nigra* L. | UOG-000505 | Tree | Dicot |
| 44 | Salvicaceae | *Azolla pinnata* R. Br. | UOG-000454 | Herb | Pteridophytes |
| 45 | Solanaceae | *Datura alba* L. | UOG-000394 | Shrub | Dicot |
|  |  | *Nicotiana plumbaginifolia* Viv. | UOG-000445 | Herb | Dicot |
|  |  | *Physalis minima* L. | UOG-000502 | Herb | Dicot |
|  |  | *Solanum xanthocarpum* L. | UOG-000508 | Shrub | Dicot |
|  |  | *Solanum nigrum* L. | UOG-000395 | Herb | Dicot |
| 46 | Tamaricaceae | *Tamarix aphylla* L. | UOG-000396 | Shrub | Monocot |
| 47 | Thymelaeaceae | *Thymelia passive* Mill. | UOG-000511 | Herb | Dicot |
| 48 | Typhaceae | *Typha latifolia*L. | UOG-000396 | Shrub | Monocot |
| 49 | Utricaceae | *Urtica urens* L. | UOG-000516 | Herb | Dicot |
| 50 | Verbenaceae | *Lantana camara* L. | UOG-000398 | Shrub | Dicot |
|  |  | *Verbena officinalis* L. | UOG-000518 | Herb | Dicot |
| 51 | Zygophyllaceae | *Tribulus terrestris* L. | UOG-000399 | Herb | Dicot |
